# Supplementary figures and images for: Order, please! Uncertainty in the ordinal-level classification of Chlorophyceae
Source: PeerJ. 2019 May 15;7:e6899. doi: 10.7717/peerj.6899 (PMC6525593; doi:10.7717/peerj.6899)

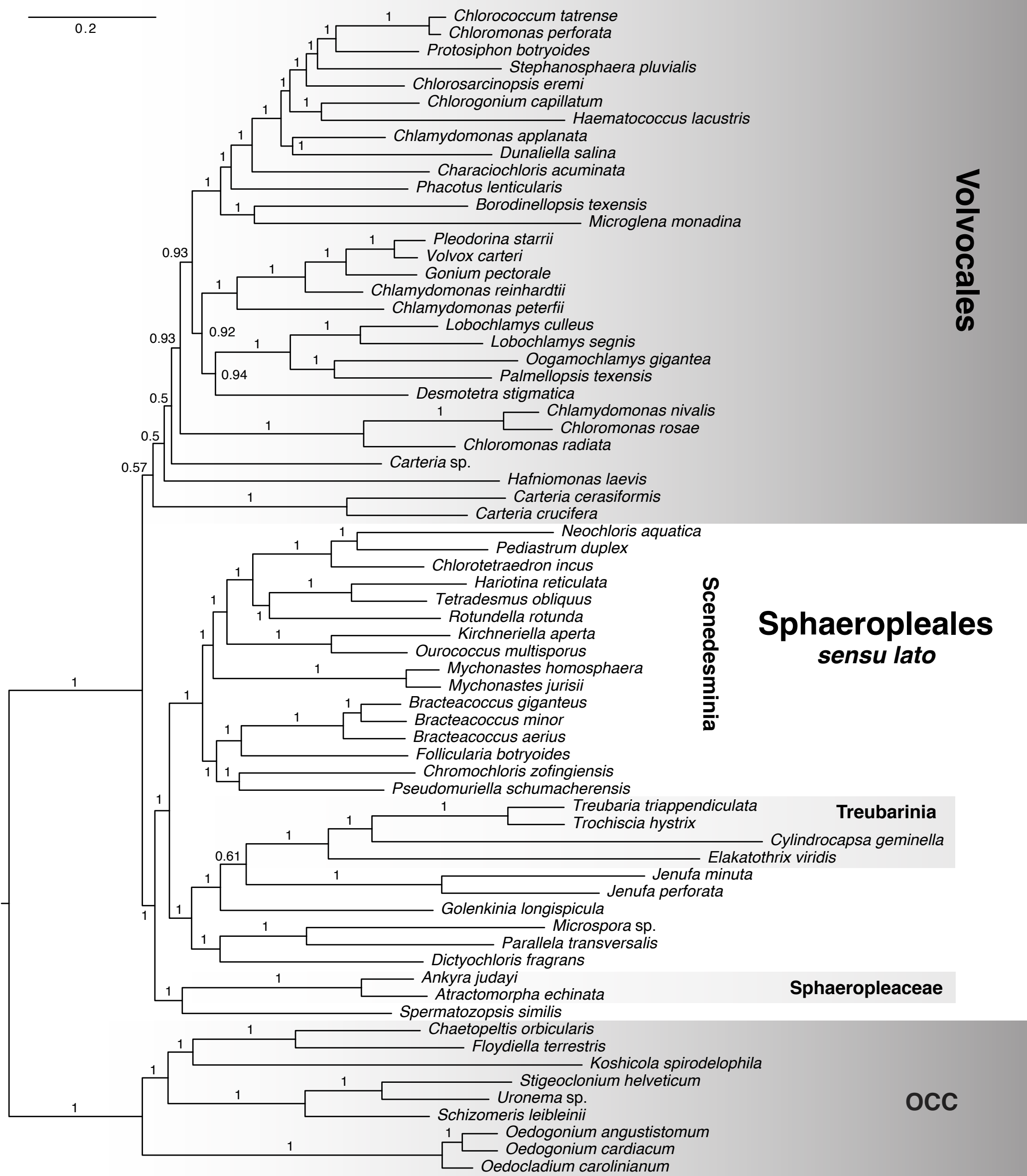

Supplement: Figure S1 — Scale bar indicates the expected number of substitutions per site. [file peerj-07-6899-s001.pdf]

SVDQuartets bootstrap consensus

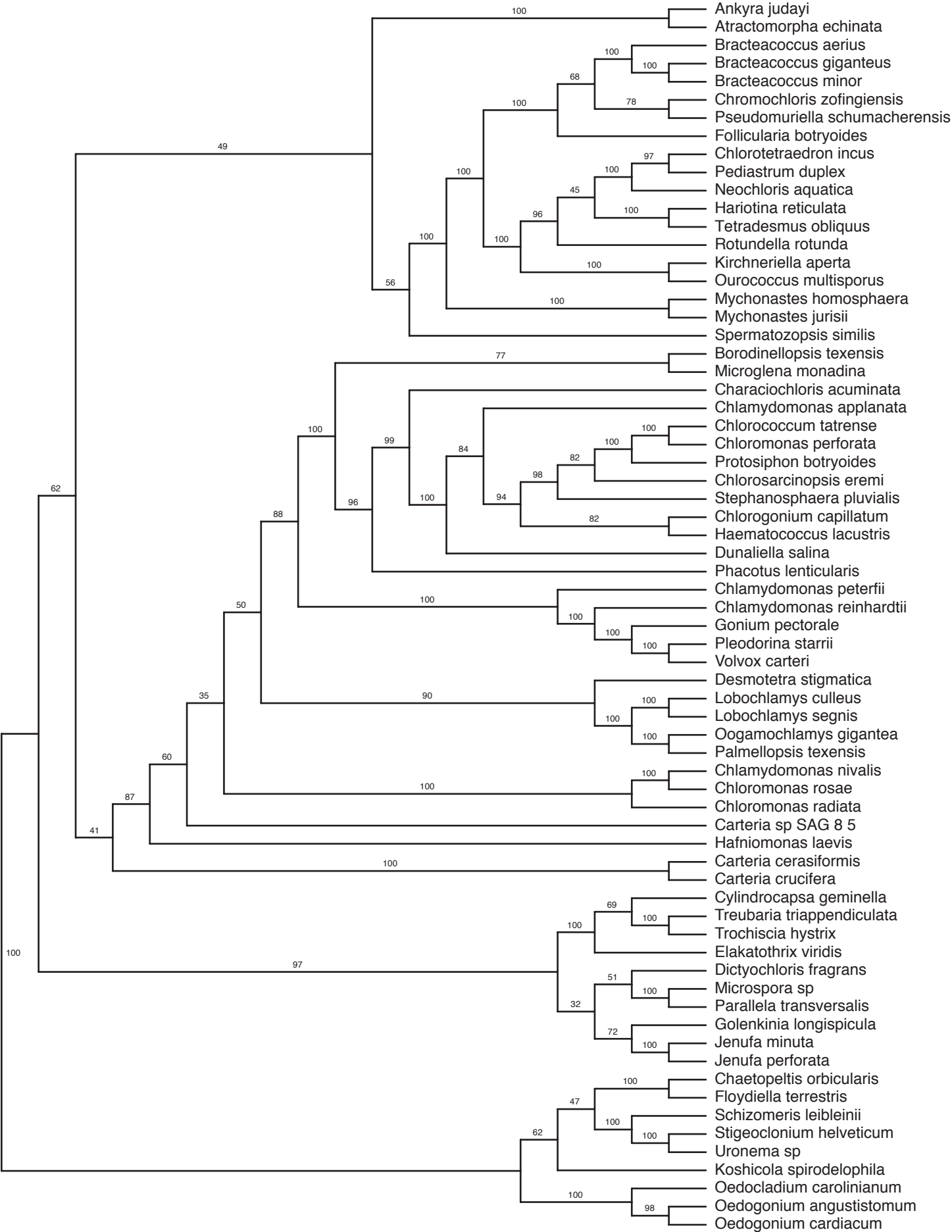

Supplement: Figure S2 [file peerj-07-6899-s002.pdf]

0.009

**Volvocales**

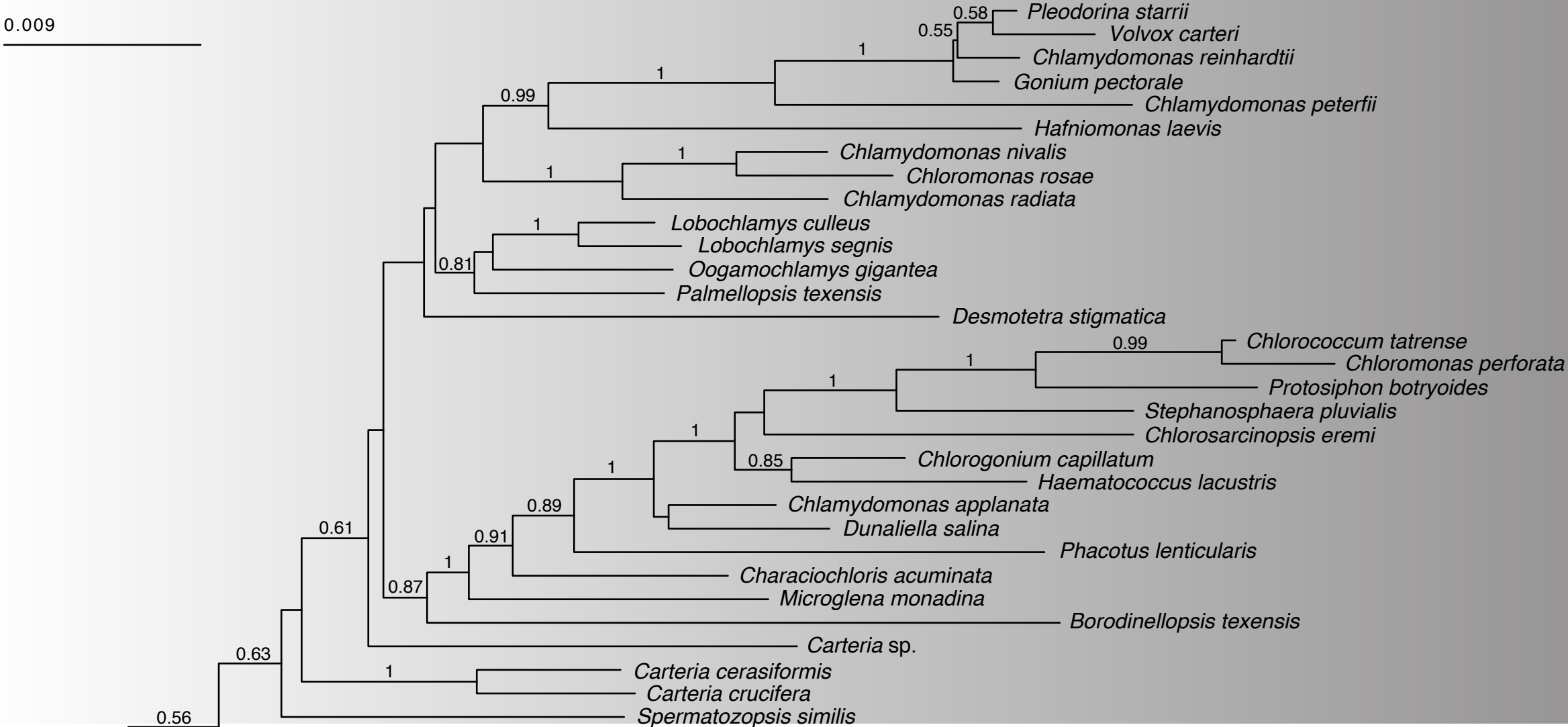

**Treubarinia**

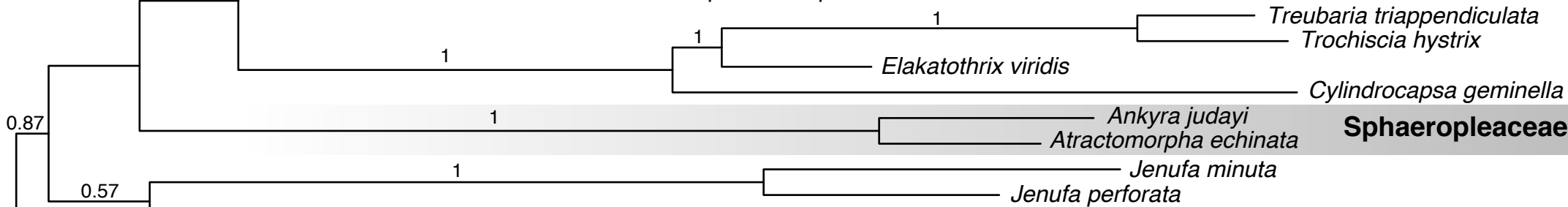

**Sphaeropleaceae**

**Scenedesminia**

**Microsporaceae**

**OCC**

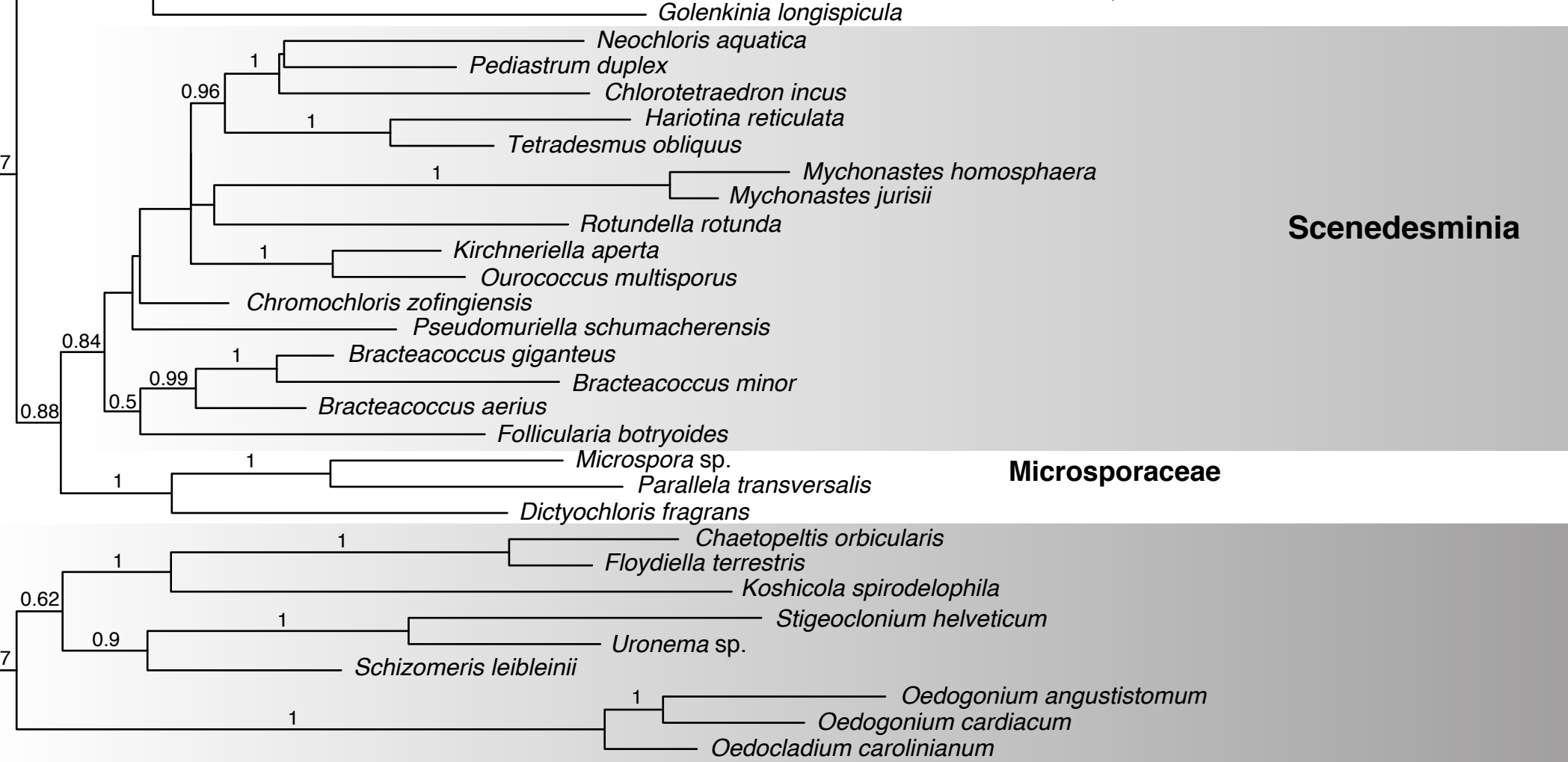

Supplement: Figure S3 — Scale bar indicates the expected number of substitutions per site. [file peerj-07-6899-s003.pdf]

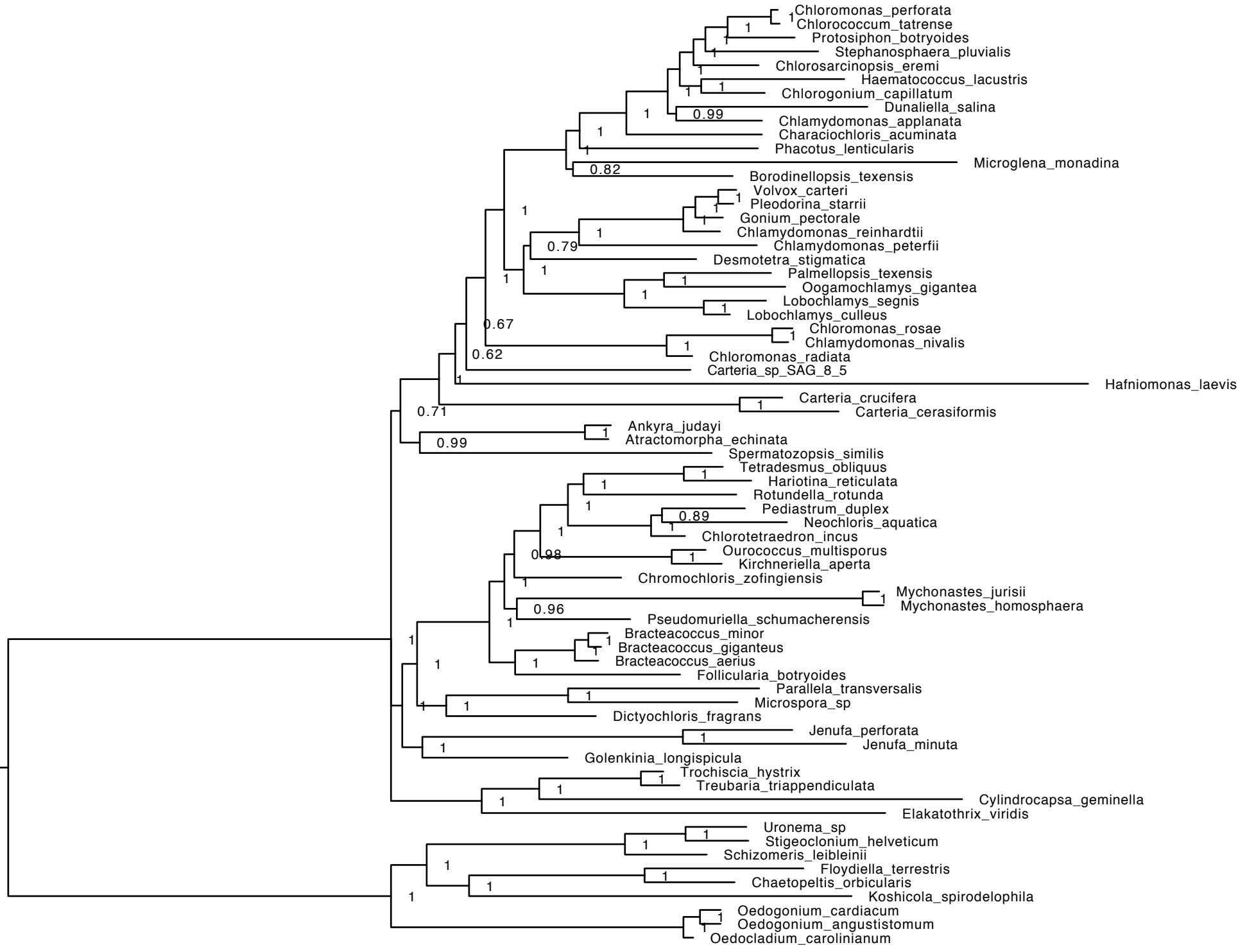

0.09

Supplement: Figure S4 — Scale bar indicates the expected number of substitutions per site. [file peerj-07-6899-s004.pdf]

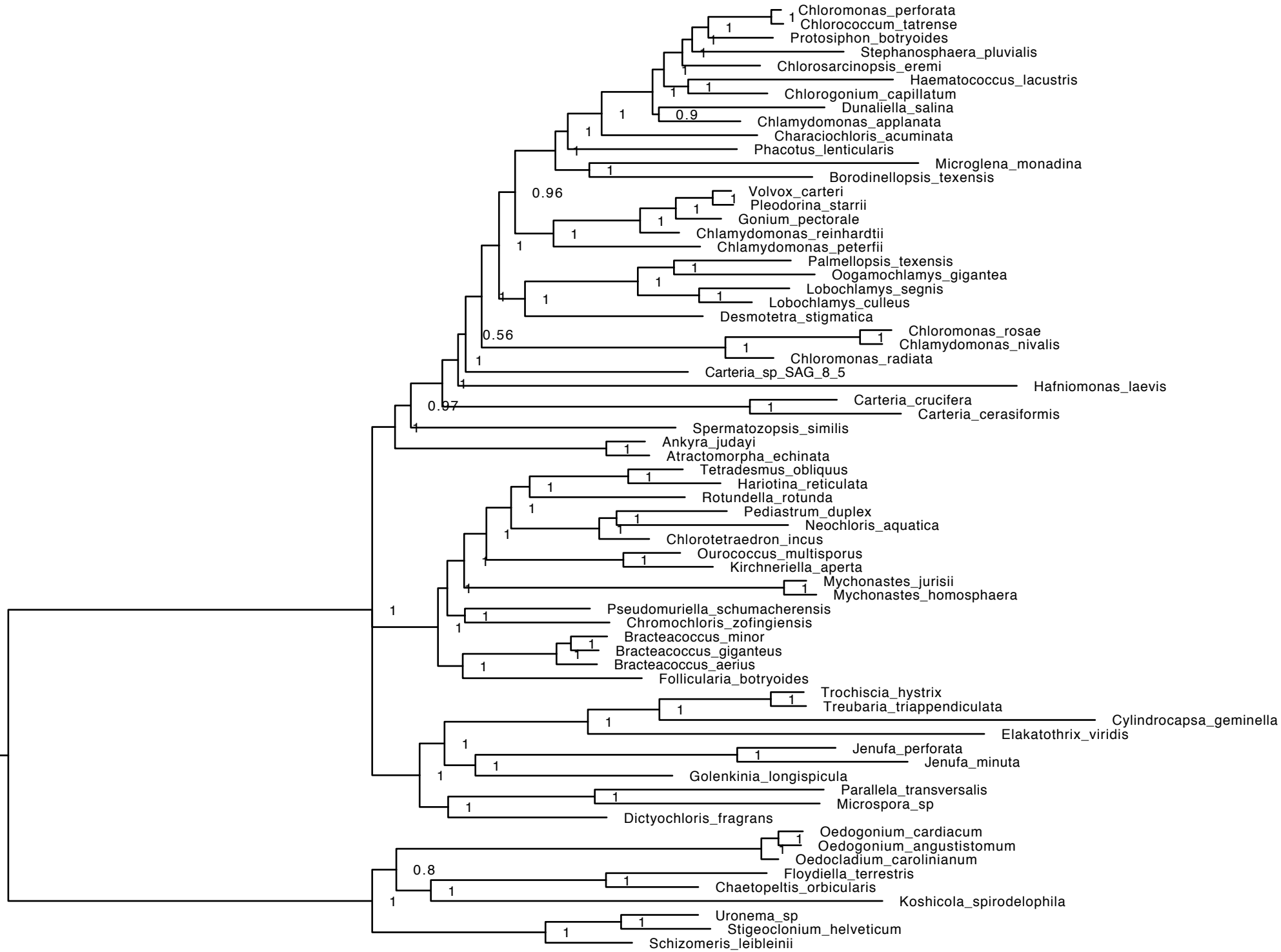

0.3

Supplement: Figure S5 — Scale bar indicates the expected number of substitutions per site. [file peerj-07-6899-s005.pdf]

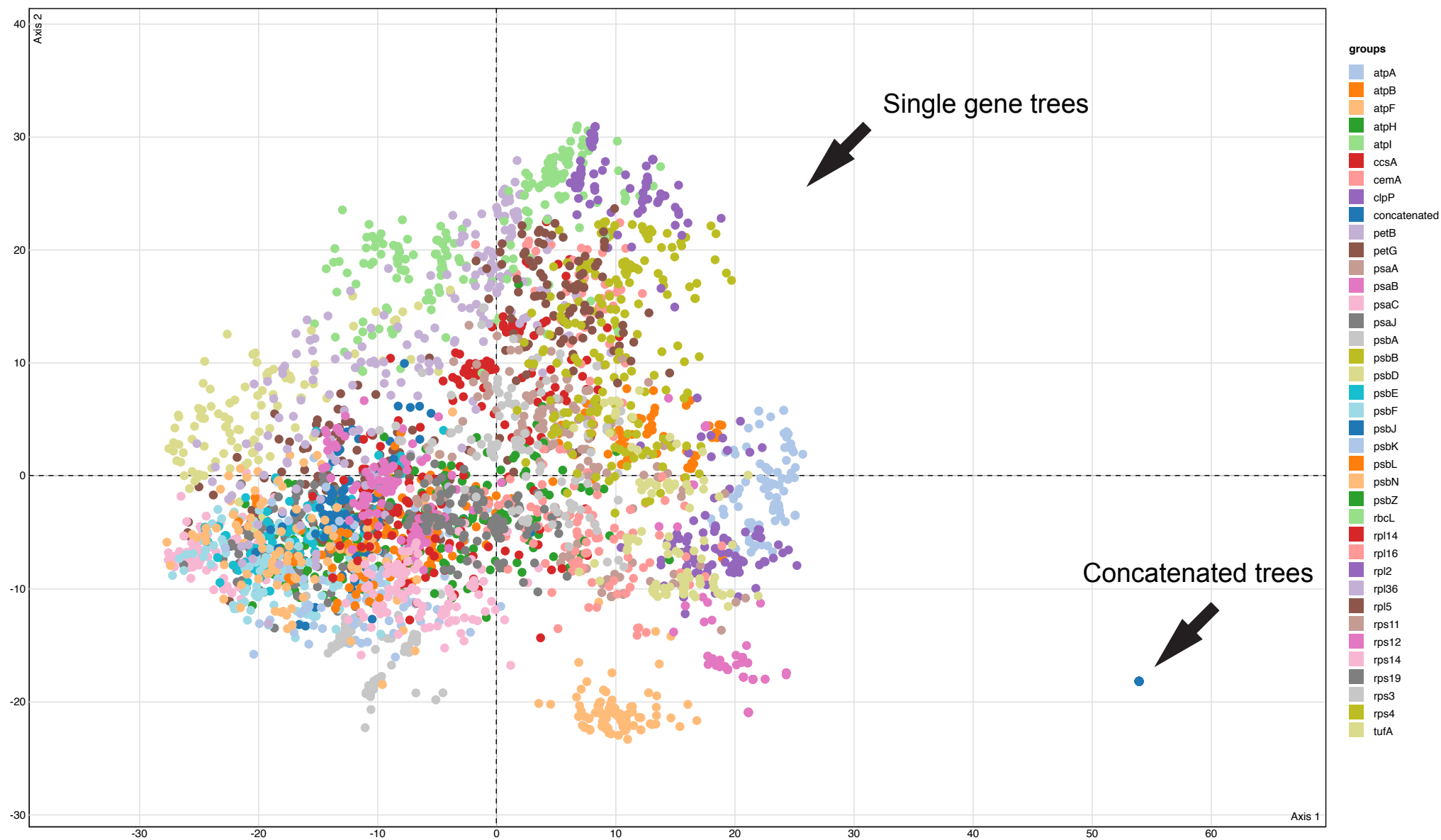

Supplement: Figure S6 — Two-dimensional scatter plot based on Treespace analysis of distances among 100 posterior trees from each of 37 single-gene Bayesian analyses (colors correspond to genes) and including the concatenated analysis (blue, marked with arrow). This plot specifically focuses on Sphaeropleales s. l.; the remaining files and scripts can be found in the Supplementary Alignments and Trees folder. [file peerj-07-6899-s006.pdf]

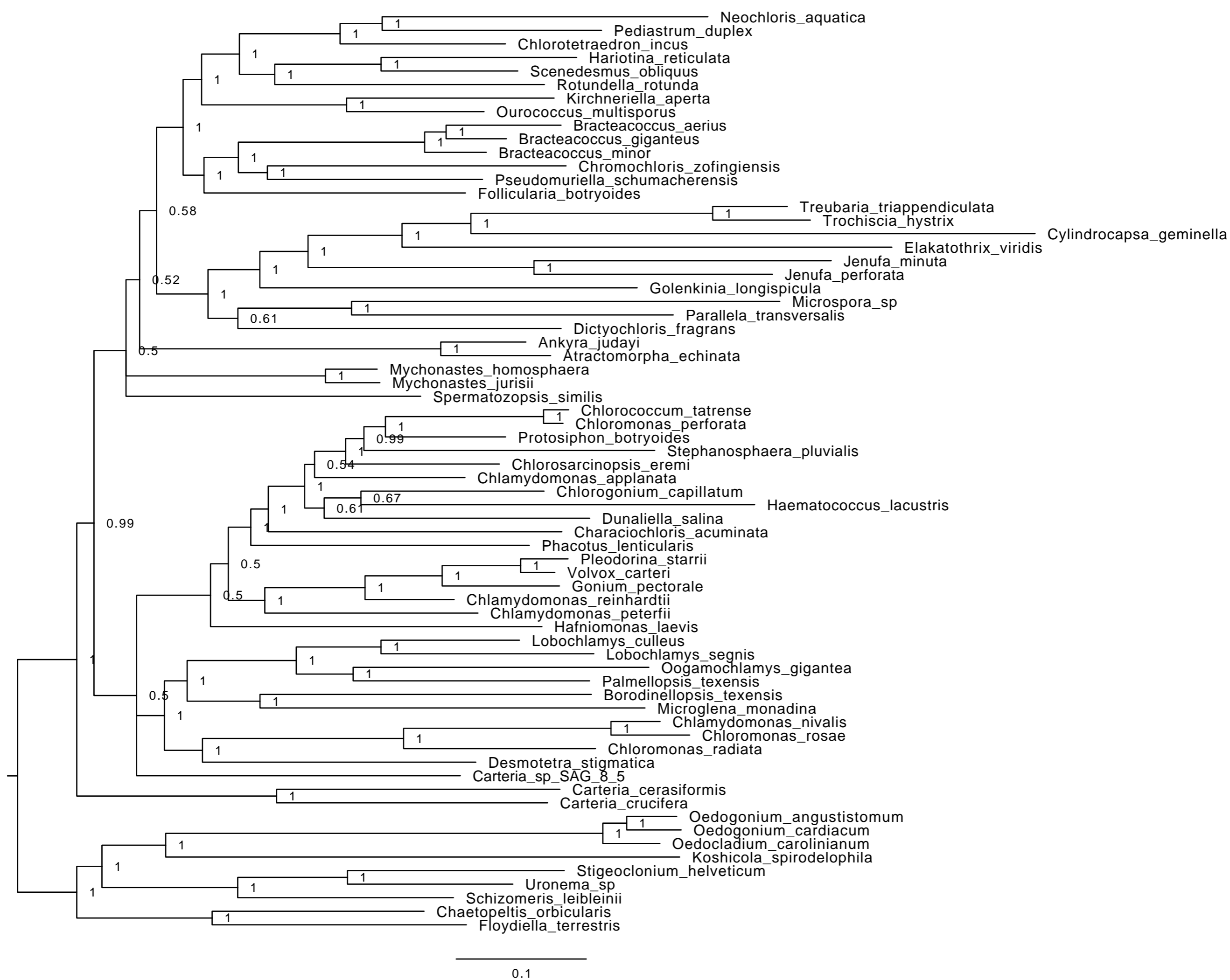

Supplement: Supplemental Information 1 — This compressed folder contains untrimmed, masked alignments for individual chloroplast genes (subfolder nexus), as well as trimmed alignments with appropriate analysis blocks and the consensus trees resulting from these single-gene and concatenated analyses (MrBayes subfolders). Supplementary methods with associated references are included in the SupplementaryMethods.txt file. SVDquartets, BaliPhy, PhyloBayes and RAxML analysis files are in separate subfolders, with their respective resulting tree files. Files associated with MrBayes analysis of the 1st and 2nd positions and the analysis of the 3rd positions are placed in the subfolders 1st_and_2nd_positions_only and 3rd_positions_only, respectively. The 18Splastid_combined subfolder contains the MrBayes analysis materials (alignment and consensus tree) for the analysis including nucleotide plastid data as well as 18S nucleotide data. Documentation, trees and scripts used to create Fig. 3 are included in the “Figure3” subfolder, and documentation for and full results of Treespace analyses are in the “Treespace” subfolder. The results of the AU topology tests are included in a Word document. [file peerj-07-6899-s008.zip › supplementary_alignments_trees/3rd_positions_only/3rdpositions.pdf]

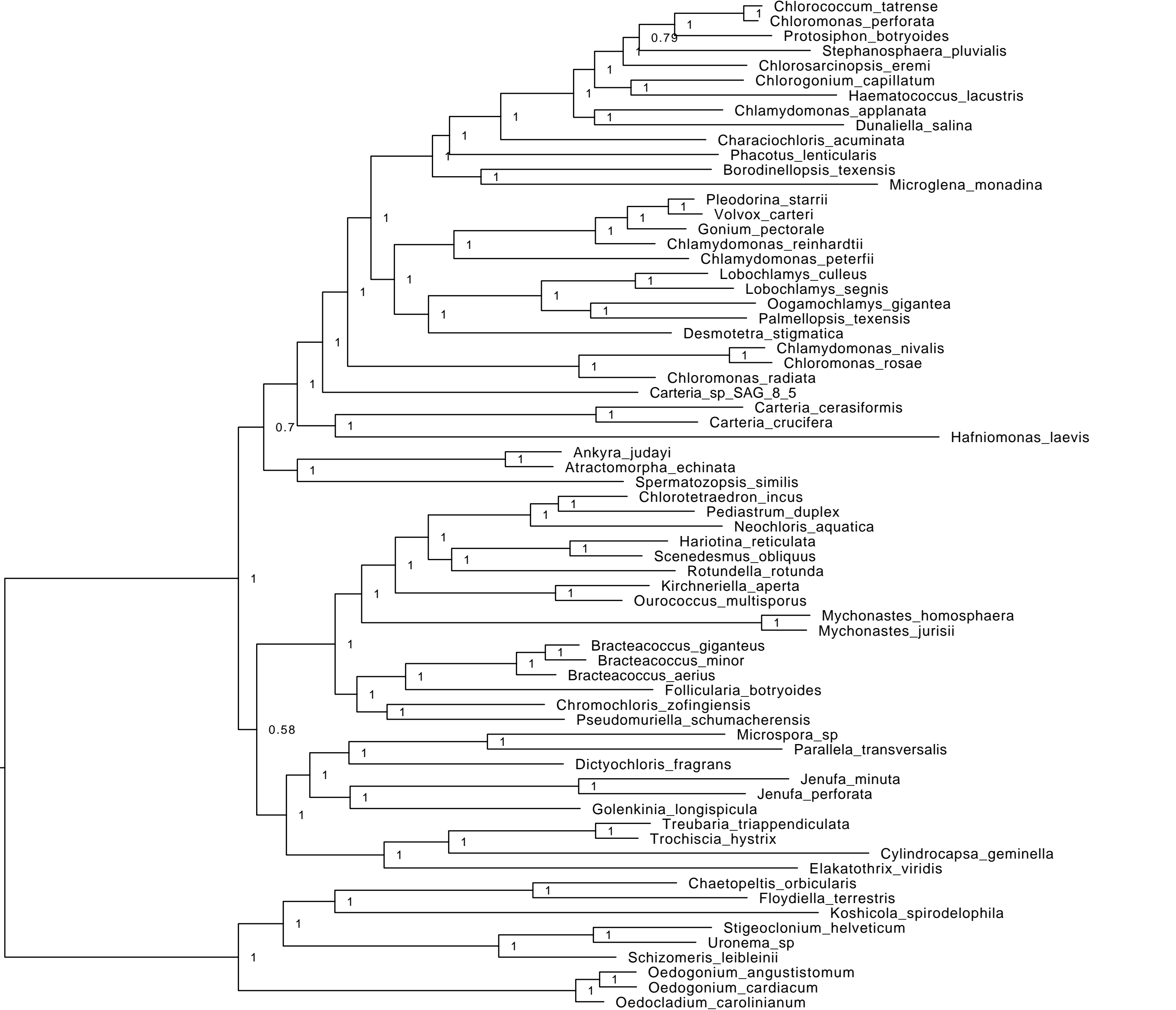

0.06

Supplement: Supplemental Information 1 — This compressed folder contains untrimmed, masked alignments for individual chloroplast genes (subfolder nexus), as well as trimmed alignments with appropriate analysis blocks and the consensus trees resulting from these single-gene and concatenated analyses (MrBayes subfolders). Supplementary methods with associated references are included in the SupplementaryMethods.txt file. SVDquartets, BaliPhy, PhyloBayes and RAxML analysis files are in separate subfolders, with their respective resulting tree files. Files associated with MrBayes analysis of the 1st and 2nd positions and the analysis of the 3rd positions are placed in the subfolders 1st_and_2nd_positions_only and 3rd_positions_only, respectively. The 18Splastid_combined subfolder contains the MrBayes analysis materials (alignment and consensus tree) for the analysis including nucleotide plastid data as well as 18S nucleotide data. Documentation, trees and scripts used to create Fig. 3 are included in the “Figure3” subfolder, and documentation for and full results of Treespace analyses are in the “Treespace” subfolder. The results of the AU topology tests are included in a Word document. [file peerj-07-6899-s008.zip › supplementary_alignments_trees/1st_and_second_positions_only/1st2ndpositions.pdf]
