# Supplementary material for: Order, please! Uncertainty in the ordinal-level classification of Chlorophyceae
Source: PeerJ. 2019 May 15;7:e6899. doi: 10.7717/peerj.6899 (PMC6525593; doi:10.7717/peerj.6899)
Supplement: Supplemental Information 1 — This compressed folder contains untrimmed, masked alignments for individual chloroplast genes (subfolder nexus), as well as trimmed alignments with appropriate analysis blocks and the consensus trees resulting from these single-gene and concatenated analyses (MrBayes subfolders). Supplementary methods with associated references are included in the SupplementaryMethods.txt file. SVDquartets, BaliPhy, PhyloBayes and RAxML analysis files are in separate subfolders, with their respective resulting tree files. Files associated with MrBayes analysis of the 1st and 2nd positions and the analysis of the 3rd positions are placed in the subfolders 1st_and_2nd_positions_only and 3rd_positions_only, respectively. The 18Splastid_combined subfolder contains the MrBayes analysis materials (alignment and consensus tree) for the analysis including nucleotide plastid data as well as 18S nucleotide data. Documentation, trees and scripts used to create Fig. 3 are included in the “Figure3” subfolder, and documentation for and full results of Treespace analyses are in the “Treespace” subfolder. The results of the AU topology tests are included in a Word document. [file peerj-07-6899-s008.zip › supplementary_alignments_trees/Results of AU topology test.docx]

Results of Shimodaira Approximately Unbiased (AU) tests for differences among topologies, based on 10000 bootstrap replicates. For each data set, two alternative topologies were tested. Within columns are the -lnL scores corresponding to each topology using that specific data set. The AU tests were done under the GTR+I+gamma model for the nucleotide data set, and the LG+I+gamma model for the amino acid data set, with parameter values estimated from the data prior to the AU tests. Boldface font indicates the best tree. Topologies marked by “*” are significantly different.

|  | Data set | |
| --- | --- | --- |
| topology | Concatenated plastid nucleotide (Fig. 2a) | Concatenated plastid amino acid (Fig. 2b) |
| Concatenated plastid nucleotide (Fig. 2a) | **878882.40197** | 238206.02947* |
| Concatenated plastid amino acid (Fig. 2b) | 879859.37477* | **238064.11712** |

* p<0.05

In summary, each data set supports ‘its own’ topology over the alternative, and the difference is statistically significant in both cases.
