# Supplementary material for: Order, please! Uncertainty in the ordinal-level classification of Chlorophyceae
Source: PeerJ. 2019 May 15;7:e6899. doi: 10.7717/peerj.6899 (PMC6525593; doi:10.7717/peerj.6899)
Supplement: Supplemental Information 1 — This compressed folder contains untrimmed, masked alignments for individual chloroplast genes (subfolder nexus), as well as trimmed alignments with appropriate analysis blocks and the consensus trees resulting from these single-gene and concatenated analyses (MrBayes subfolders). Supplementary methods with associated references are included in the SupplementaryMethods.txt file. SVDquartets, BaliPhy, PhyloBayes and RAxML analysis files are in separate subfolders, with their respective resulting tree files. Files associated with MrBayes analysis of the 1st and 2nd positions and the analysis of the 3rd positions are placed in the subfolders 1st_and_2nd_positions_only and 3rd_positions_only, respectively. The 18Splastid_combined subfolder contains the MrBayes analysis materials (alignment and consensus tree) for the analysis including nucleotide plastid data as well as 18S nucleotide data. Documentation, trees and scripts used to create Fig. 3 are included in the “Figure3” subfolder, and documentation for and full results of Treespace analyses are in the “Treespace” subfolder. The results of the AU topology tests are included in a Word document. [file 18Splastid.con.pdf › supplementary_alignments_trees/18Splastid_combined/18Splastid.con.pdf]

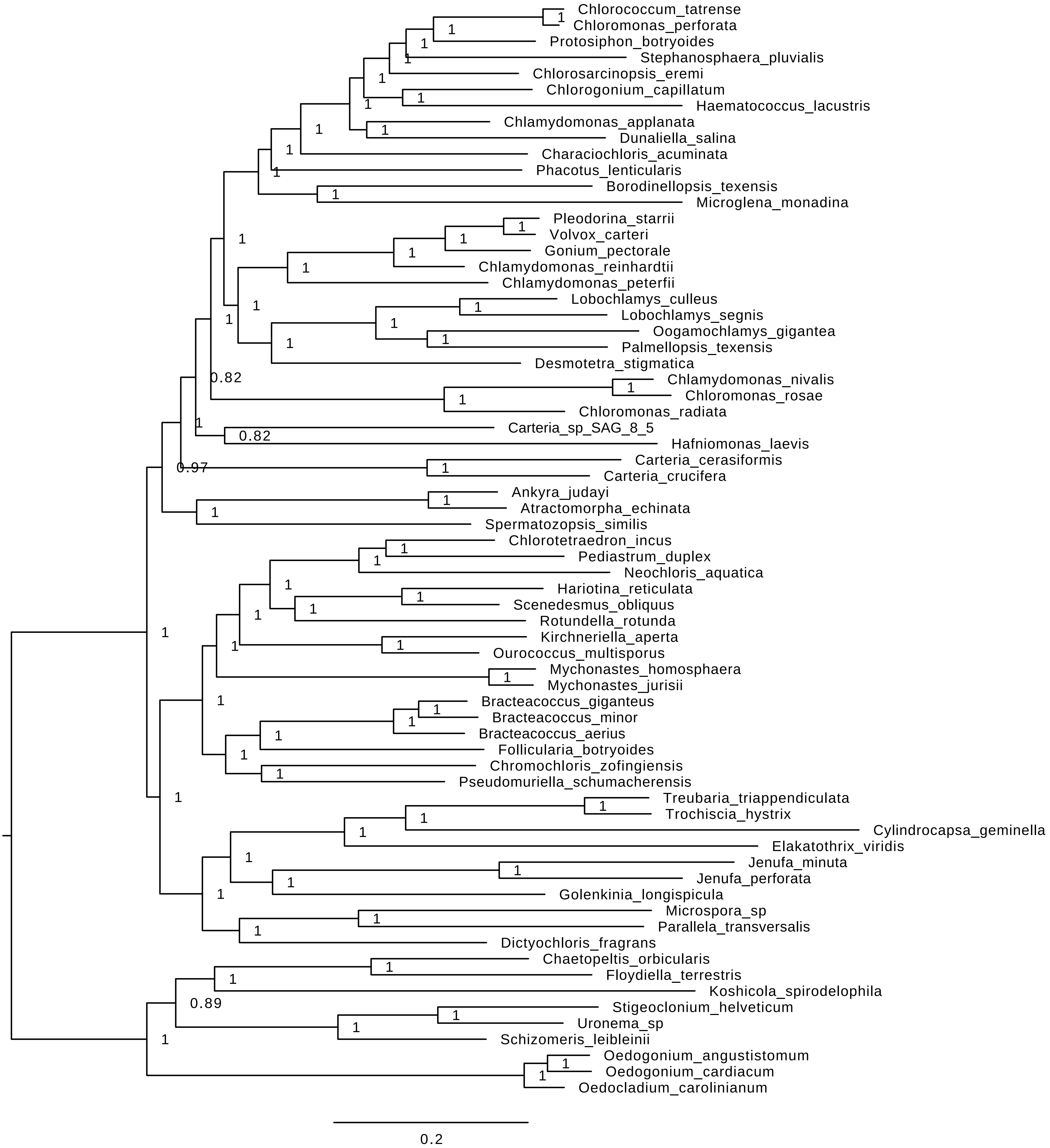
Chlorococcum_tatrense
Chloromonas_perforata
Protosiphon_botryoides
Stephanosphaera_pluvialis
Chlorosarcinopsis_eremi
Chlorogonium_capillatum
Haematococcus_lacustris
Chlamydomonas_applanata
Dunaliella_salina
Characiochloris_acuminata
Phacotus_lenticularis
Borodinellopsis_texensis
Microglena_monadina
Pleodorina_starrii
Volvox_carteri
Gonium_pectorale
Chlamydomonas_reinhardtii
Chlamydomonas_peterfii
Lobochlamys_culleus
Lobochlamys_segnis
Oogamochlamys_gigantea
Palmellopsis_texensis
Desmotetra_stigmatica
Chlamydomonas_nivalis
Chloromonas_rosae
Chloromonas_radiata
Carteria_sp_SAG_8_5
Hafniomonas_laevis
Carteria_cerasiformis
Carteria_crucifera
Ankyra_judayi
Atractomorpha_echinata
Spermatozopsis_similis
Chlorotetraedron_incus
Pediastrum_duplex
Neochloris_aquatica
Hariotina_reticulata
Scenedesmus_obliquus
Rotundella_rotunda
Kirchneriella_aperta
Ourococcus_multisporus
Mychonastes_homosphaera
Mychonastes_jurisii
Bracteacoccus_giganteus
Bracteacoccus_minor
Bracteacoccus_aerius
Follicularia_botryoides
Chromochloris_zofingiensis
Pseudomuriella_schumacherensis
Treubaria_triappendiculata
Trochiscia_hystrix
Cylindrocapsa_geminella
Elakatothrix_viridis
Jenufa_minuta
Jenufa_perforata
Golenkinia_longispicula
Microspora_sp
Parallela_transversalis
Dictyochloris_fragrans
Chaetopeltis_orbicularis
Floydiella_terrestris
Koshicola_spirodelophila
Stigeoclonium_helveticum
Uronema_sp
Schizomeris_leibleinii
Oedogonium_angustistomum
Oedogonium_cardiacum
Oedocladium_carolinianum
0.82
0.82
0.97
0.89
0.2
