# Supplementary material for: Order, please! Uncertainty in the ordinal-level classification of Chlorophyceae
Source: PeerJ. 2019 May 15;7:e6899. doi: 10.7717/peerj.6899 (PMC6525593; doi:10.7717/peerj.6899)
Supplement: Supplemental Information 1 — This compressed folder contains untrimmed, masked alignments for individual chloroplast genes (subfolder nexus), as well as trimmed alignments with appropriate analysis blocks and the consensus trees resulting from these single-gene and concatenated analyses (MrBayes subfolders). Supplementary methods with associated references are included in the SupplementaryMethods.txt file. SVDquartets, BaliPhy, PhyloBayes and RAxML analysis files are in separate subfolders, with their respective resulting tree files. Files associated with MrBayes analysis of the 1st and 2nd positions and the analysis of the 3rd positions are placed in the subfolders 1st_and_2nd_positions_only and 3rd_positions_only, respectively. The 18Splastid_combined subfolder contains the MrBayes analysis materials (alignment and consensus tree) for the analysis including nucleotide plastid data as well as 18S nucleotide data. Documentation, trees and scripts used to create Fig. 3 are included in the “Figure3” subfolder, and documentation for and full results of Treespace analyses are in the “Treespace” subfolder. The results of the AU topology tests are included in a Word document. [file peerj-07-6899-s008.zip › supplementary_alignments_trees/Treespace/Materials_Methods.docx]

We used TREESPACE (Jombart et al., 2017) to visualize the variability of posterior trees among genes in 2-to 3-dimensional Euclidean space. In this approach, the pairwise distances between posterior trees were computed (as Robinson Foulds unweighted metric, Robinson and Foulds 1981) from the package phangorn, and decomposed into a low-dimensional Euclidean space using metric multidimensional scaling (MDS). Before applying MDS, TREESPACE transformed the unweighted Robinson Foulds tree distances into Euclidean distances using Cailliez’s transformation (Cailliez, 1983). The analysis was performed on posterior trees from 37 genes comprising entire set of Chlorophycean taxa (68 spp.) sampled in the study. TREESPACE was performed on the collections 3800 posterior trees (representing 100 randomly sampled trees/gene) obtained from MrBayes MCMC analysis.

References:

Cailliez, F. (1983). The analytical solution of the additive constant problem. *Psychometrika*, 48, 305–308.

Robinson, D. F., & Foulds, L. R. (1981). Comparison of phylogenetic trees.

*Mathematical Biosciences*, 53, 131–147.

Jombart, T. *et al*. (2017) TREESPACE: Statistical exploration of landscapes of phylogenetic trees. *Mol. Ecol. Resour.,* https://doi.org/10.1111/1755-0998.

.
