# Supplementary material for: Order, please! Uncertainty in the ordinal-level classification of Chlorophyceae
Source: PeerJ. 2019 May 15;7:e6899. doi: 10.7717/peerj.6899 (PMC6525593; doi:10.7717/peerj.6899)
Supplement: Supplemental Information 1 — This compressed folder contains untrimmed, masked alignments for individual chloroplast genes (subfolder nexus), as well as trimmed alignments with appropriate analysis blocks and the consensus trees resulting from these single-gene and concatenated analyses (MrBayes subfolders). Supplementary methods with associated references are included in the SupplementaryMethods.txt file. SVDquartets, BaliPhy, PhyloBayes and RAxML analysis files are in separate subfolders, with their respective resulting tree files. Files associated with MrBayes analysis of the 1st and 2nd positions and the analysis of the 3rd positions are placed in the subfolders 1st_and_2nd_positions_only and 3rd_positions_only, respectively. The 18Splastid_combined subfolder contains the MrBayes analysis materials (alignment and consensus tree) for the analysis including nucleotide plastid data as well as 18S nucleotide data. Documentation, trees and scripts used to create Fig. 3 are included in the “Figure3” subfolder, and documentation for and full results of Treespace analyses are in the “Treespace” subfolder. The results of the AU topology tests are included in a Word document. [file peerj-07-6899-s008.zip › supplementary_alignments_trees/Treespace/grouping_1/2_3.pdf]

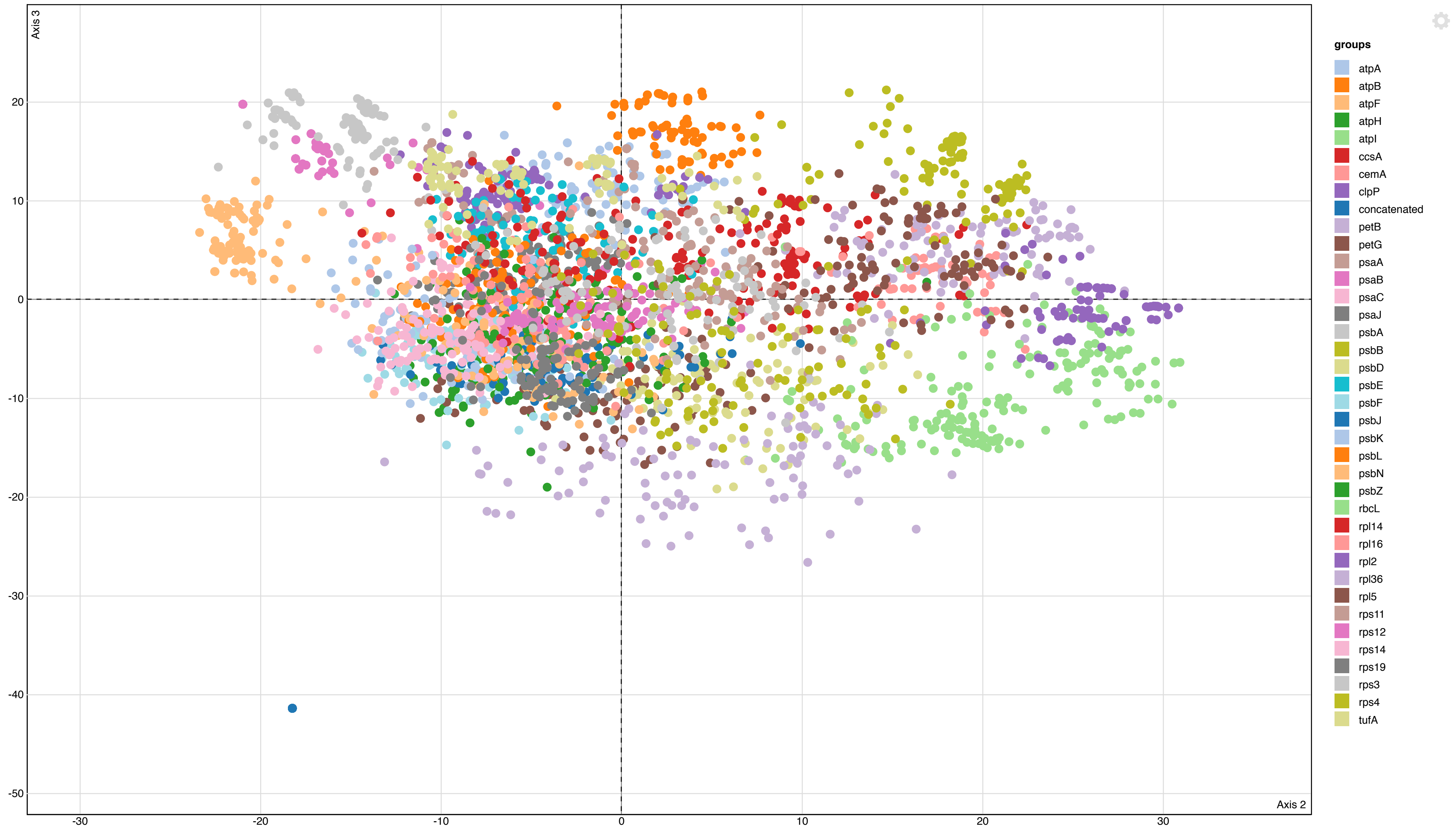

Axis 3
Axis 2
20
10
0
-10
-20
-30
-40
-50
-30
-20
-10
0
10
20
30
groups
atpA
atpB
atpF
atpH
atpI
ccsA
cemA
clpP
concatenated
petB
petG
psaA
psaB
psaC
psaJ
psbA
psbB
psbD
psbE
psbF
psbJ
psbK
psbL
psbN
psbZ
rbcL
rpl14
rpl16
rpl2
rpl36
rpl5
rps11
rps12
rps14
rps19
rps3
rps4
tufA
